# Influence of dietary supplementation with *Bacillus licheniformis* and *Saccharomyces cerevisiae* as alternatives to monensin on growth performance, antioxidant, immunity, ruminal fermentation and microbial diversity of fattening lambs

**DOI:** 10.1038/s41598-018-35081-4

**Published:** 2018-11-12

**Authors:** Peng Jia, Kai Cui, Tao Ma, Fan Wan, Wenyi Wang, Dong Yang, Yunfei Wang, Baolin Guo, Lifang Zhao, Qiyu Diao

**Affiliations:** 1 Key Laboratory of Feed Biotechnology, Ministry of Agriculture and Rural Affairs, Feed Research Institute, Chinese Academy of Agricultural Sciences, Beijing, 100081 China; 2 Scientific Research Institute of Agricultural and Animal Husbandry in Bayannaoer, Inner Mongolia Bayannaoer, Beijing, 015000 China; 3 Beijing Xindayang Technological Co., Ltd, Beijing, 100081 China

## Abstract

Alternatives to antibiotics for improving productivity and maintaining the health of livestock health are urgently needed. The scope of this research was conducted to investigate the effects of two alternatives (*Bacillus licheniformis* and *Saccharomyces cerevisiae*) to monensin on growth performance, antioxidant capacity, immunity, ruminal fermentation and microbial diversity of fattening lambs. One hundred and sixty Dorper × Thin-tailed Han sheep (32 ± 3.45 kg BW) were randomly assigned into 5 treatments of n = 32 lambs/group. Lambs in the control group were fed a basal diet (NC) while the other four treatments were fed basal diets supplemented with monensin (PC), *Bacillus licheniformis* (BL), *Saccharomyces cerevisiae* (SC), and the combination of *Bacillus licheniformis* and *Saccharomyces cerevisiae* with protease (BS), respectively. The experiment lasted for 66 d. Feed intake was recorded every 2 d and lambs were weighed every 20 d. Ten lambs from each group were slaughtered at the end of the trial, and samples of serum and rumen fluid were collected. The results indicated that the dietary regimen did not affect the dry matter intake (DMI). The average daily gain (ADG) of BS treatment was significantly higher than NC group (*P* < 0.05). Compared with the NC treatment, the other four supplementation treatments increased the concentration of growth hormone (GH), insulin-like growth factor I (IGF-I) and insulin (INS) (*P* < 0.05). The malondialdehyde (MDA) and total antioxidant capacity (TAOC) showed no significant difference among the 5 treatments while the activity of superoxide dismutase (SOD) and glutathione peroxidase (GSH-Px) of BS group was significantly increased (*P* < 0.05). The supplementation regimen decreased the concentration of ammonia Nitrogen (NH_3_-N) and increased the content of microbial crude proteins (MCP) (*P* < 0.05). The supplementation of antibiotics and probiotics reduced the concentrations of acetate and increased the concentrations of propionate (*P* < 0.05). The supplementation treatments increased the relative abundance of *Lentisphaerae*, *Fibrobacteres* and *Tenericutes* at the phylum level, whereas at the genus level, they increased the relative abundance of *Fibrobacter* (*P* < 0.05). Overall, this study confirmed the facilitating effect of *B*. *licheniformis*, *S*. *cerevisiae* and their compounds on growth performance, improve the antioxidant capacity and immune function, and beneficially manipulate ruminal fermentation and microbial diversity of fatting lambs.

## Introduction

The application of feed additives in livestock ration has an immense importance for improving nutrient utilization and productivity in the animal system. Monensin is a common feed additive which is associated the benefits of improving the efficiency of energy metabolism and rumen fermentation^1^. However, the use of antibiotics as a livestock feed additive is banned gradually due to the risk of accumulation of residues in animal products and the emergence of antibiotics-resistant bacterial strains^2–4^. Growing interest has focused on probiotics as alternatives of antibiotics which is characteristic of improving animal productive performance, inhibiting the colonization of pathogenic microorganisms and maintaining the balance of microflora of the digestive tract of the host^5–7^.

Numerous studies have described the role of *Bacillus* which has the probiotic characteristics of improving microbial balance, digestive processes and immunity of hosts. Research conducted on the influence of *B*. *subtilis* on ruminants showed that *B*. *subtilis* altered the rumen fermentation pattern of calves and increased the milk yield of ewes^8^. Studies of *B*. *subtilis natto* supplementation indicated that it was effective in increasing lactation performance of early lactation dairy cows possibly by altering the rumen fermentation pattern^9^. Qiao *et al*. reported that *B*. *licheniformis* increased ruminal apparent nutrient digestibility of neutral detergent fibre, acid detergent fibre, and organic matter while *B*. *subtilis* had no significant effect on rumen fermentation characteristics, duodenal microbial N flow and ruminal apparent nutrient digestibility^8^. To our knowledge, research on application of *Bacillus* in fattening lambs is scarce.

Supplementation of yeast is confirmed having the function of stabilizing rumen pH, increasing volatile fatty acids and decreasing ammonia concentration^10^. Various researches have been proposed to explain the effects that live yeast supplementation strains may have on rumen fermentation and ruminant production. The most commonly used strain of yeast is *Saccharomyces cerevisiae*, which can help to increase the digestibility of nutrients, improve the weight gain and feed conversion rate and modulate the composition of microbial ecosystem^11^. But still the results on the use of *S*. *cerevisiae* in ruminants are contradictory that in many cases no influence or opposing results were obtained. Part of the study revealed that *S*. *cerevisiae* supplemented diets did not improve growth performances of East-Friesian or Malpura lambs in fattening stage. The number of anaerobic and aerobic rumen bacteria was not affected by *S*. *cerevisiae* supplementation^12^.

Variable response to feeding *B*. *licheniformis* or *S*. *cerevisiae* in ruminant production emphasizes the need for understanding the actual mechanisms through which probiotics exert their functions. Considering the limited research relating to the combine effects of *B*. *licheniformis* and *S*. *cerevisiae* on microbiota, more insight into the mode of action of them is needed. The objective of this study was to investigate the effect of *B*. *licheniformis*, *S*. *cerevisiae* and combination of them on growth performance, antioxidant capacity, immunity, ruminal fermentation and microbial diversity of fattening lamb.

## Results

### Growth performance

The average daily gain (ADG), dry matter intake (DMI) and feed efficiency (FCR, DMI/ADG) were analyzed to determine the effects of *B*. *licheniformi*, *S*. *cerevisiae* and their combination on growth performance of lambs (Table 1). The IBW and DMI showed no difference among the five treatments. The ADG of lambs in PC, BL, SC and BS group showed no significant difference, while the ADG of lambs in the BS group was significantly higher than that in NC group (*P* < 0.05). The FCR of the BL group and BS group were significantly lower than that in NC group (*P* < 0.05).

**Table 1:** Effects of dietary supplementation with *B*. *licheniformis* and *S*. *cerevisiae* as alternatives to monensin on growth performance of fattening lambs.

Items	Treatments					SEM	*P*-value
	NC	PC	BL	SC	BS		
Initial bodyweight (kg)	32.29	32.02	32.28	32.34	32.47	0.29	0.995
dry matter intake (g/d)	1601.70	1592.94	1584.24	1612.95	1616.23	14.92	0.968
Average daily gain (g/d)	265.65^b^	295.81^ab^	304.66^ab^	297.85^ab^	322.26^a^	4.77	0.047
Feed conversion ratio	6.03^a^	5.52^ab^	5.21^b^	5.42^ab^	5.03^b^	0.11	0.038

^a,b^Values in the same row with different superscripts differ significantly (*P* < 0.05).

### Serum profiles

Compared with the NC and PC group, the serum concentration of GH and IGF-1 of lambs in SC and BS group were significantly increased (*P* < 0.05). Serum INS concentration of the lambs in BL and BS groups was higher than that in the NC group (*P* < 0.05). The TAOC and MDA showed no significant difference among the five groups, while the supplementation of *B*. *licheniformis* and *S*. *cerevisiae* increased the activity of SOD and GSH-Px. Lambs supplemented with *B*. *licheniformis*, *S*. *cerevisiae* and the compounds showed an increased serum concentration of IgA and IgG (*P* < 0.05) (Table 2).

**Table 2:** Effects of dietary supplementation with *B*. *licheniformis* and *S*. *cerevisiae* as alternatives to monensin on serum profiles of fattening lambs.

Items	Treatments					SEM	*P*-value
	NC	PC	BL	SC	BS		
GH (ng/mL)	1.21^d^	1.22^cd^	1.32^bc^	1.35^b^	1.47^a^	0.02	<0.0001
IGF-1 (μg/mL)	29.49^c^	30.64^c^	30.88^c^	34.49^b^	36.29^a^	0.46	<0.0001
INS (μIU/mL)	18.12^b^	19.13^ab^	21.61^a^	19.11^ab^	21.73^a^	0.45	0.028
T-AOC (U/ml)	9.47	9.47	9.55	9.62	9.78	0.04	0.130
MDA (nmol/mL)	5.19	5.53	5.54	5.52	5.36	0.07	0.409
SOD (U/ml)	100.06^b^	96.16^b^	95.25^b^	96.19^b^	121.50^a^	2.55	0.002
GSH-PX (μmol/L)	846.26^b^	847.53^b^	855.65^b^	860.01^ab^	870.99^a^	2.51	0.007
IGA (μg/mL)	0.43^b^	0.52^ab^	0.59^a^	0.58^a^	0.60^a^	0.02	0.002
IGG (μg/mL)	15.98^c^	17.65^bc^	19.91^b^	19.19^b^	24.40^a^	0.59	<0.0001
IGM (μg/mL)	0.96^c^	1.25^bc^	1.30^b^	1.26^bc^	1.62^a^	0.05	0.002

^a,b^Values in the same row with different superscripts differ significantly (*P* < 0.05).

### Rumen fermentation

No difference of pH value was detected among the five groups. The concentration of NH_3_-N of lambs in NC group was significantly higher than that of the other four groups (*P* < 0.05). Compared to the NC group, dietary biological agent regimen significantly increased the MCP content of lamb (*P* < 0.05). No difference of MCP and TVFA were detected among the four supplementary treatments. The lambs supplemented with the monensin, *B*. *licheniformis*, *S*. *cerevisiae* or compounds had a lower percentage of acetate and higher percentage of propionate than that of the NC group (*P* < 0.05). The butyrate percentage of lambs in SC group was significantly higher than that in the other four groups while no obvious difference was detected among PC, BL and BS groups (Table 3).

**Table 3:** Effects of dietary supplementation with *B*. *licheniformis* and *S*. *cerevisiae* as alternatives to monensin on ruminal fermentation of fattening lambs.

Items	Treatments					SEM	*P*-value
	NC	PC	BL	SC	BS		
pH	6.71	6.72	6.79	6.76	6.75	0.02	0.763
NH3-N (mg/dL)	27.01^a^	23.01^b^	22.73^b^	22.07^b^	21.59^b^	0.54	0.007
MCP (mg/dL)	41.13^b^	44.89^ab^	51.41^a^	49.05^ab^	53.97^a^	1.49	0.043
Total VFA (mol/dl)	44.35	47.87	46.70	48.74	48.89	0.67	0.186
Acetate (%)	67.64^a^	62.30^b^	61.75^b^	61.03^b^	61.11^b^	0.44	<0.0001
Propionate (%)	16.36^b^	20.07^a^	20.47^a^	20.42^a^	21.00^a^	0.27	<0.0001
Butyrate (%)	6.84^c^	8.22^b^	7.98^b^	9.06^a^	8.59^ab^	0.15	<0.0001
A:P	4.16^a^	3.11^b^	3.02^b^	3.01^b^	2.91^b^	0.08	<0.0001

^a,b^Values in the same row with different superscripts differ significantly (*P* < 0.05).

### Sequencing depth and index of Microbial Community

**Figure 1:**
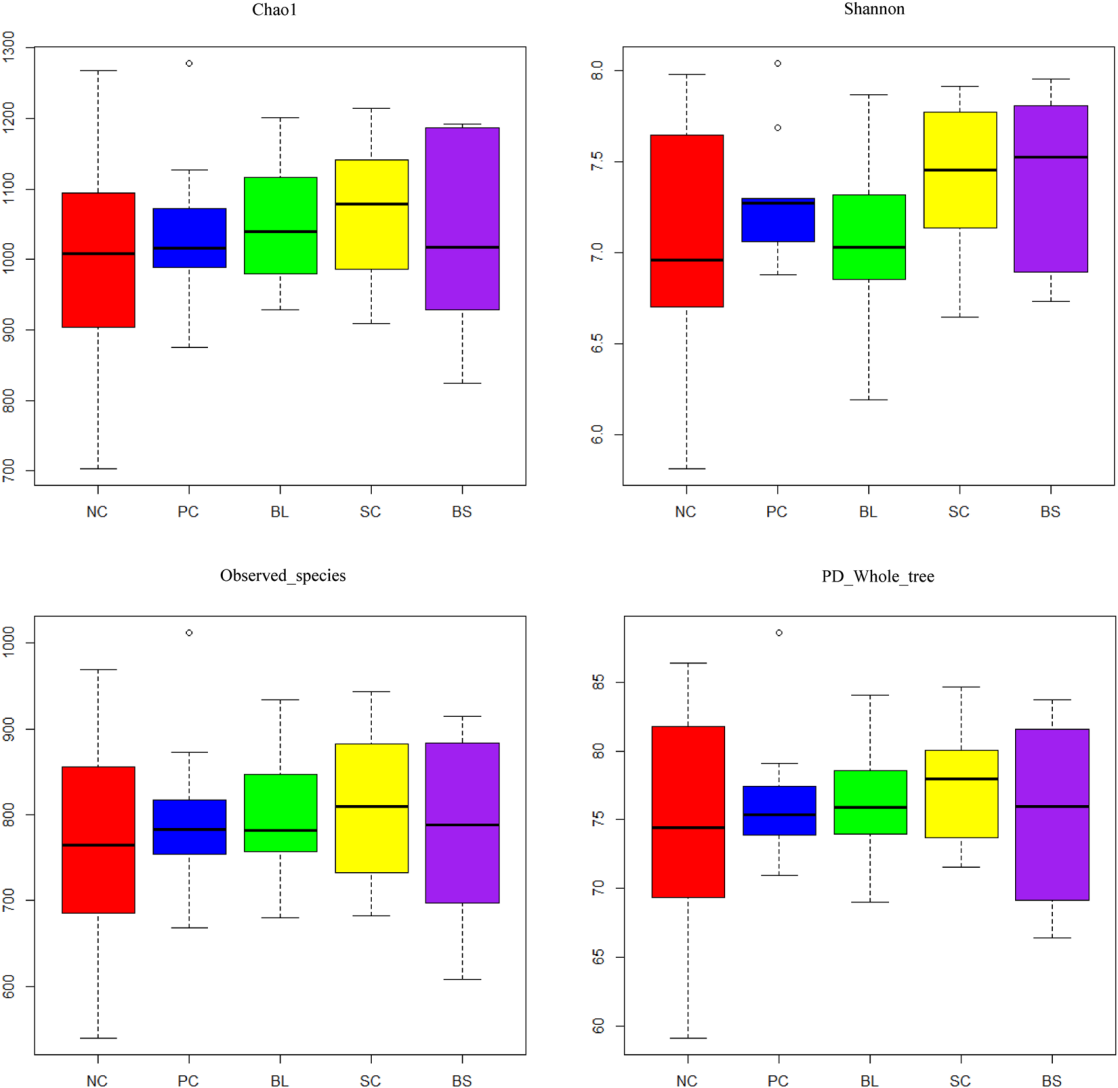
Community richness estimates and diversity indices for different treatments

Illumina HiSeq sequencing generated a total of 2,328,167 clean reads with an average of 46,563 ± 20,780 reads per sample after data filtering, quality control, and removal of primers, chimeras, and low-confidence singletons. All reads were classified into 2,052 operational taxonomic units (OTUs) based on ≥97% nucleotide sequence identity between reads. The OTU numbers of NC, PC, BL, SC and BS was 971, 1026, 1034, 1038 and 1018, respectively. The α diversity index analysis was shown in Fig. 1. The community richness estimates and diversity indices showed no significant difference among the five groups. Principal coordinates analysis plots based on unweighted UniFrac distance metrics revealed difference of microbial diversity among the treatments (Fig. S1).

### Bacterial composition across different treatments

**Figure 2:**
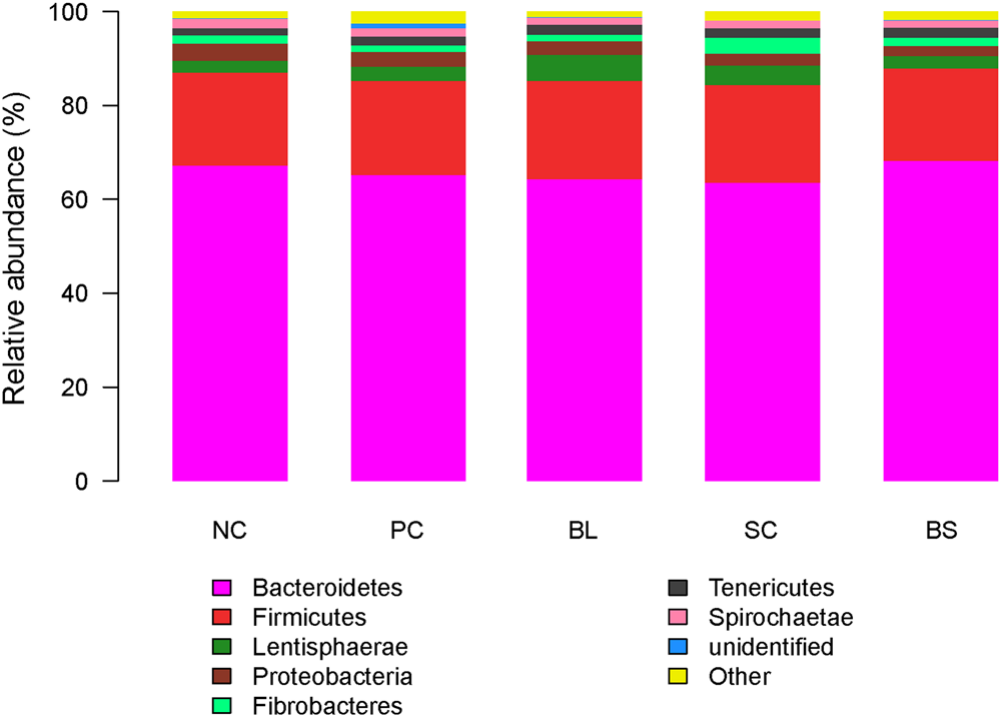
Phylum level composition. Color-coded bar plot showing the relative abundances of the abundant phyla across different treatments.

All sequences were classified from phylum to species based on the SILVA taxonomic database. At the phylum level, 21 phyla were detected in the samples from the rumen fluid of lamb (Table 4, Fig. 2). The major sequences obtained from the five groups belonged to *Bacteroidet*es, *Firmicutes*, *Lentisphaerae* and *Proteobacteria*. However, the relative abundance of these predominant phyla varied considerably among the different calf groups. Within the CON treatment, *Bacteroidetes* (67.09%), *Firmicutes* (19.87%) and *Proteobacteria* (3.83%) were the dominant phyla while the other treatments were dominated by *Bacteroidetes*, *Firmicutes* and *Lentisphaerae*. The supplementation of *B*. *licheniformis*, and *S*. *cerevisiae* increased the percentage of *Lentisphaerae* and *Tenericutes* (*P* < 0.10). Compared to the other four treatments, the percentage of *Fibrobacteres* of SC treatment showed an increased tendency (*P* < 0.10).

**Table 4:** Comparison of the dominant phylum (average relative abundance ≥1% for at least one treatment) within the rumen. ^a,b^Values in the same row with different superscripts differ significantly (*P* < 0.05).

Phylum	Treatments					SEM	*P*-value
	NC	PC	BL	SC	BS		
*Bacteroidetes*	67.09	65.61	65.80	63.52	68.41	0.82	0.420
*Firmicutes*	19.87	20.03	20.31	20.99	19.33	0.44	0.828
*Lentisphaerae*	2.64	2.98	5.15	4.34	2.70	0.33	0.051
*Proteobacteria*	3.83	2.97	2.72	2.68	2.32	0.31	0.627
*Fibrobacteres*	1.59	1.45	1.18	2.85	1.64	0.20	0.080
*Tenericutes*	1.43	1.86	2.11	2.03	2.23	0.09	0.061
*Spirochaetae*	2.05	1.79	1.52	1.59	1.60	0.09	0.410

**Figure 3:**
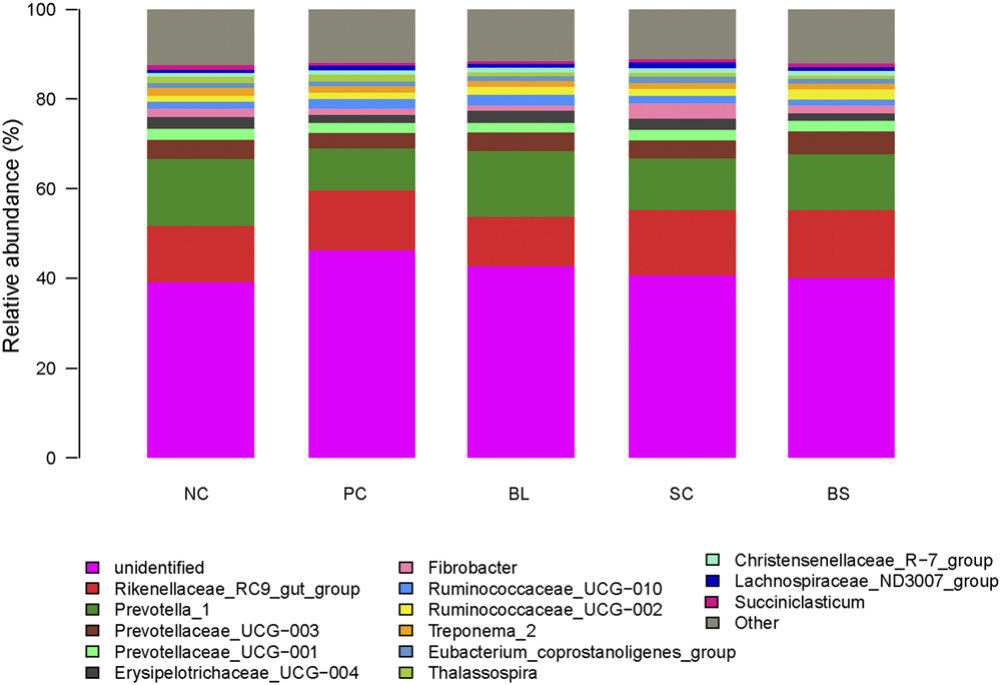
Genus level composition. Color-coded bar plot showing the relative abundances of different genera across different treatments.

**Figure 4:**
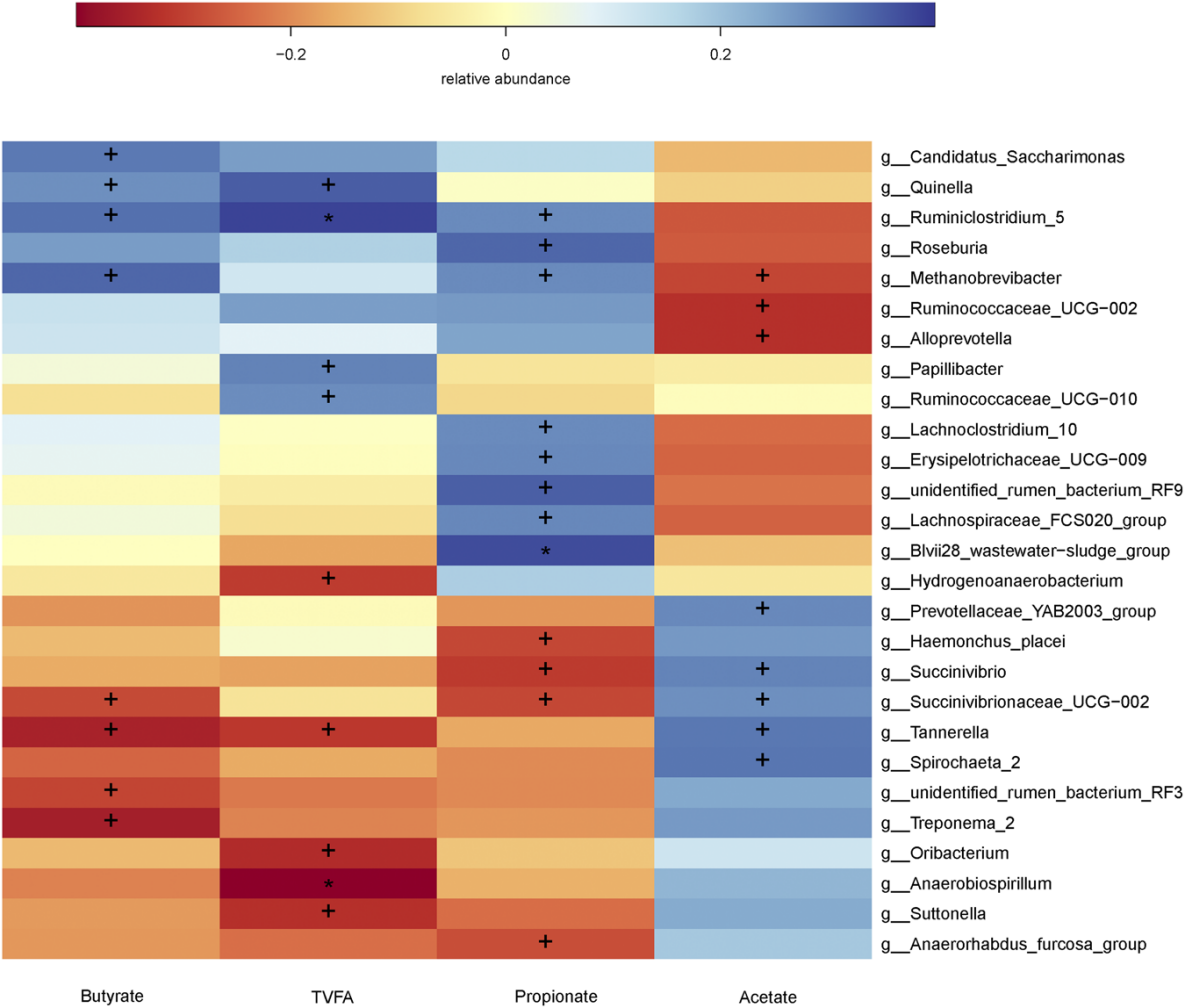
Spearman correlation analysis of VFA and microbiome at genus level. The depth of the color indicates the correlation between species and environmental factors. The “+” and “*” indicates the different level at 0.05 and 0.01, respectively.

At the genus level, 123 genera belonging to the 21 phyla were detected in the samples. In general, 29 most abundant shared genera with a relative abundance ≥1% were present in all samples across different treatments, and the relative abundance levels were markedly different among the five treatments (Table 5, Fig. 3). The major sequences obtained from the five treatments belonged to *Rikenellaceae_RC9_gut_group*, the taxa derived from *Prevotellaceae* (family), *Erysipelotrichaceae_UCG-004* and *Ruminococcaceae_UCG-010*. Compared to the other four treatments, the supplementation of *S*. *cerevisiae* significantly increased the abundance of *Fibrobacter* (*P* < 0.05). We evaluated the correlation between the ruminal fermentation parameters and bacteria at genus level by performing spearman correlation analysis. Almost 27 genera belonging to 6 phyla were exhibited significant correlation with TVFA, acetic, butyrate, and propionic (*P* < 0.05) (Fig. 4).

**Table 5:** Comparison of the dominant genus (average relative abundance ≥1% for at least one treatment) within the rumen. ^a,b^Values in the same row with different superscripts differ significantly (*P* < 0.05).

Phylum	Genus	Treatments					SEM	*P*-value
		NC	PC	BL	SC	BS		
*Bacteroidetes*	*Rikenellaceae_RC9_gut_group*	13.01	13.68	11.40	14.29	15.10	0.53	0.227
	*Prevotella_1*	14.61	9.87	14.31	11.09	13.32	0.86	0.337
	*Prevotellaceae_UCG-003*	4.10	3.57	4.24	3.76	4.79	0.29	0.740
	*Prevotellaceae_UCG-001*	2.30	2.27	2.08	2.24	2.43	0.14	0.967
*Firmicutes*	*Erysipelotrichaceae_UCG-004*	2.75	1.805	2.72	2.02	1.61	0.29	0.626
	*Ruminococcaceae_UCG-010*	1.69	1.95	2.19	1.62	1.37	0.14	0.433
	*Ruminococcaceae_UCG-002*	1.37	1.49	1.63	1.79	2.23	0.16	0.467
	*Eubacterium_coprostanoligenes _group*	1.25	1.04	1.03	1.48	1.06	0.10	0.537
	*Lachnospiraceae_ND3007_group*	0.82	0.98	0.83	1.46	0.76	0.12	0.341
	*Christensenellaceae_R-7_group*	0.81	0.86	1.01	1.12	1.03	0.06	0.408
	*Succiniclasticum*	1.03	0.61	0.55	0.70	0.76	0.07	0.190
*proteobacteria*	*Thalassospira*	1.46	1.46	0.70	0.84	0.78	0.25	0.784
*Firbrobacteres*	*Fibrobacter*	1.57^b^	1.44^b^	1.16^b^	2.84^a^	1.63^b^	0.19	0.042
*Spirochaetes*	*Treponema_2*	1.71	1.49	1.30	1.32	1.32	0.08	0.493

## Discussion

Studies of antibiotics in animal husbandry proved the benefits in terms of efficiency in the gain of body weight and reduction in the occurrence of subclinical diseases^13,14^. As an ionophore antibiotic, monensin has been added to high grain rations as feed additives for approximately four decades based on its ability to alter fermentation pattern, to promote growth, and to decrease the occurrence of subacute ruminal acidosis^15,16^. Antibiotic alternatives such as *B*. *licheniformis*, *S*. *cerevisiae* and plant extract have captured the interest of researchers because of the rise in antimicrobial resistance. In the current study, the supplementation of monensin, *B*. *licheniformis*, *S*. *cerevisiae* and the combination of *B*. *licheniformis* and *S*. *cerevisiae* with protease improved the growth performance and feed conversion ratio without affecting the DMI of lambs, but no significant differences were detected between the supplementation treatments. Our findings are in agreement with those of previous reports that probiotics supplementation and monensin were equally effective to increase the weight gain of lambs and no significant difference of DMI was detected^1,17,18^.

Growth Hormone (GH) is a peptide hormone of crucial importance in stimulating the production of IGF-1 and growth of organisms. Many researches have been carried out focused on the effect of supplementation of probiotics on the hormone level and growth performance of aquatic animals. The research on the sea bass juveniles and *Oreochromis niloticus* showed that the addition of probiotics or the combination of probiotics and prebiotics elevated the serum level of GH, IGF-I and INS, and increased the gene expression of them^19,20^. In the current study, the supplementation of *S*. *cerevisiae* and the compound of *B*. *licheniformis* and *S*. *cerevisiae* significantly increased the serum level of GH and IGF1.

Numerous studies have described the role of probiotics and bioactive compounds in enhancing the immunity and antioxidation of mammals^21^. Sohail *et al*. indicated that dietary supplementation of probiotic mixture reduced the some of the detrimental effects of oxidative damage of broilers^22^. Study of necrotizing enterocolitis caused by oxidant showed that the activity of SOD and GSH-Px were up-regulated with supplementation probiotics, prebiotics and synbiotics^23^. In this research, we also found that the compound of *B*. *licheniformis* and *S*. *cerevisiae* supplementation significantly increased of the serum content of SOD and GSH-Px. Meanwhile, the compound supplementation increased the serum concentrations of IgG and IgM, which are the indicators of nonspecific humoral immune competences in ruminants. Similar results were reported by Mendieta *et al*. and Andrea *et al*. that the addition of *S*. *cerevisiae* yeast cell walls and *B*. *subtilis* to diets increased serum IgG and IgM concentrations in broilers, respectively^24,25^.

Probiotics have been reported to elevate efficient of rumen function by stabilizing rumen pH and increasing volatile fatty acids production. However, the effect of probiotic supplementation on rumen pH of small ruminants has not been clearly defined. El-Ghani *et al*. recorded an increase in rumen pH while Tripathi *et al*. found no effect in rumen pH^26,27^. What is interesting is that some researchers reported a reduction in rumen pH after supplementation with *Saccharomyces* or an equal mixture of *Saccharomyces* to growing lambs. In this research, monensin or probiotics supplementation had no effect on rumen pH but decreased the concentration of ammoniacal nitrogen and increased the content of microbial proteins. The result indicated that probiotics could improve the efficiency of rumen fermentation, which is in accordance with the research of *B*. *subtilis natto* applied in dairy cows^28^. The change of ruminal NH_3_-N and MCP level that reflects the nitrogen utilization of the rumen microbials might attribute to the higher average daily gain of lambs.

Volatile fatty acids are the primary products of rumen fermentation which contribute to rumen epithelium development of fattening sheep. However, previous studies showed inconsistent idea of the effect of probiotics on ruminal VFAs. Sadiek *et al*. and Abd El-Ghani *et al*. reported that feeding of pronifer or *S*. *cerevisiae* increased the VFA production of sheep and goats, respectively^27,29^. On the contrary, studies of growing lambs or adult goats fed on probiotic supplemented diets indicated a significant reduction in ruminal VFA formation. In the present study, the concentration of total volatile fatty acids was not influenced by either a single administration of monensin, *B*. *licheniformis*, *S*. *cerevisiae* or the blended administration of *B*. *licheniformis* and *S*. *cerevisiae*, which is consistent with the studies of lambs and goats^27,29^. However, probiotic or monensin supplemented diets decreased the concentration of acetate while the concentration of propionate and butyrate was increased. There are many reports of this change in the A:P ratio^17,30,31^. The VFA profile is associated with effects on end-product composition and energy balance in ruminants. Propionate is the main source of glucose and a substrate for gluconeogenesis for the ruminant, while acetate and butyrate are precursors for long-chain fatty acid synthesis. It is in agreement with previous reports that high glucogenic to nonglucogenic VFA ratio is beneficial for growth of finishing cattle.

Ruminants and ruminal microorganisms have evolved together for millions of years and the addition of some probiotics to the diet has been reported to improve feed utilization and growth performance of the lamb by enhancing of rumen microbial ecosystem^32^. In this research, HiSeq sequencing of 16S rRNA was used to evaluate the changes in the ruminal bacterial community of lambs under different biological agents in diets. The relative abundance of *Bacteroidetes* and *Firmicutes* accounted for over 84% among the five treatments, which is in agreement with previous reports that most of the bacterial community of mammals is affiliated with the phyla of *Bacteroidetes* and *Firmicutes*^33^. The richness of phylum *Lentisphaerae*, *Fibrobacteres* and *Tenericutes* was varied among the treatments, which indicated that the supplementation of *B*. *licheniformis* or *S*. *cerevisiae* affected the distribution of bacteria in the rumen. Several studies have shown that *S*. *cerevisiae* is able to stimulate lactate utilizing bacteria thus stabilizing ruminal pH and favouring fibre degradation. In this research, *S*. *cerevisiae* supplementation was increased the genus level of *Fibrobacter* but had no effect on ruminal pH among the five treatments which was consistent with the previous studies^32^. However, the other genera with a relative abundance above 1% showed no significant difference among the five treatments. Previous studies reported the potential of probiotics in manipulation of gastrointestinal microbiota; however, their efficacies often vary and are inconsistent. Numerous factors, such as the type and level of probiotics, ingredient composition of the experimental diet, and management constraints, have been shown to markedly affect the structure and activities of microbial communities in livestock animals.

## Conclusion

This study demonstrated that dietary of *B*. *licheniformis*, *S*. *cerevisiae* and their compounds improved the growth performance of fattening sheep and could be the alternatives of monensin. It also highlights that supplementation of *S*. *cerevisiae* and the compound of *B*. *licheniformis* and *S*. *cerevisiae* significantly increased the serum level of GH and IGF1, improved the antioxidant capacity and immune function of lambs. It was discovered that *B*. *licheniformis*, *S*. *cerevisiae* and their compounds increased the nitrogen utilization of the rumen microbials and changed the fermentation pattern which was beneficial for growth of fattening sheep. Based on 16 S rRNA gene sequencing method, this study indicated that supplementation of *S*. *cerevisiae* increased the genus level of *Fibrobacter*. Overall, this study confirmed the effect of *B*. *licheniformis*, *S*. *cerevisiae* and their compounds on growth performance, antioxidant capacity, immune function, ruminal fermentation and microbial diversity of fattening lambs which are of great importance for the targeted improvement of nutrient levels in ruminants.

## Materials and Methods

### Ethics statement

The animal experiments were conducted at the Fuchuan Feed Technology Co., Ltd. Modern sheep breeding base, Inner Mongolia, China. The experimental protocol was approved by the Chinese Academy of Agricultural Sciences Animal Ethics Committee, and humane animal care and handling procedures were followed throughout the experiment.

### Animals and management conditions

One hundred and sixty lambs (Dorper × Thin-tailed Han) with an initial bodyweight of 32 ± 3.45 kg were randomly assigned into 5 treatments with 4 replicates of each treatment and 8 lambs per replicate. The treatments were as follows: 1) negative control (NC): fed a basal diet; 2) positive control (PC): fed a basal diet supplemented with monensin (21 mg/kg); 3) *B*. *licheniformis* treatment (BL): fed a basal diet supplemented with *B*. *licheniformis* (4 × 10^9^ CFU); 4) *S*. *cerevisiae* treatment (SC): fed a basal diet supplemented with *S*. *cerevisiae* (3.2 × 10^9^ CFU); 5) combined *B*. *licheniformis* and *S*. *cerevisiae* treatment (BS): fed a basal diet supplemented with both *B*. *licheniformis* (6 × 10^9^ CFU) and *S*. *cerevisiae* (4 × 10^9^ CFU) with protease, respectively.

The basal diet was formulated according to NRC and our previous research and the ingredients and chemical composition of the diet are present in Table 6^34,35^. The diets were provided in the form of pellet feed and lambs at the bodyweight of 30~40 kg were supplemented with Leymus chinensis about 20% of total feeding. The feed and water were available *ad libitum* during the 66d feeding period. Feed intake was recorded every 2 d and lambs were weighed every 20 d to calculate average daily gain (ADG), average daily feed intake (ADFI) and feed efficiency (G:F ratio). Ten lambs from each treatment were slaughtered at the end of the trial.

**Table 6:** Ingredients and nutrient composition of the diets (dry matter basis).

Items	Diet	
	30~40 kg	40~50 kg
**Ingredients**		
Corn	24.00	35.00
Wheat bran	8.00	5.00
Soybean meal	4.00	5.00
Cottonseed meal	4.00	5.00
Alfalfa hay	4.00	5.00
DDGS	4.00	7.00
Bean straw	48.00	30.00
Sunflower meal	1.60	2.00
Corn protein meal	0.00	3.00
NaCl	0.40	0.50
Limestone	0.70	0.90
CaHPO_4_	0.50	0.60
Premix^a^	0.80	1.00
Total	100.0	100.0
**Nutrient levels** ^b^		
GE/(MJ/kg)	16.70	16.50
DM	93.10	92.00
CP	14.60	16.10
EE	3.23	3.35
Ash	14.00	8.71
NDF	54.40	47.50
ADF	23.90	15.00
Ca	0.74	0.75
P	0.40	0.42

^a^The premix provided the following per kg of diets: VA 15 000 IU, VD 2 200IU, VE 50 IU, Fe 55 mg, Cu 12.5 mg, Mn 47 mg, Zn 24 mg, Se 0.5 mg, I 0.5 mg, Co 0.1 mg.

^b^Nutrient levels were measured values. DM; dry matter; CP, crude protein; EE, ether extract; NDF, neutral detergent fiber; ADF, acid detergent fiber; Ca, calcium; P, phosphorus.

### Blood profiles

Blood samples were collected before morning feeding by jugular veinpuncture into one 10 ml vacutainer tubes. The samples were centrifuged (5000 g, 20 min, 4 °C) to obtain the serum, which was frozen at −20 °C until further analysis. The activity of TAOC, SOD, GSH-Px and the content of MDA were analyzed with commercial kits from Nanjing Jiancheng Bioengineering Institute (Jiangsu, China) according to the manufacturer’s protocols. Serum concentrations of IGF-1, immunoglobulin (Ig) A, M and G were detected with a microplate reader (Thermo Scientific, Multiskan GO, Waltham, MA, USA). The serum level of growth hormone and insulin was detected with radioimnunoassay kit against its specifications.

### Chemical analyses

The composition and nutrient levels of milk replacer and starters were analyzed according the official methods of analysis (Association of Official Analytical Chemists: Washington, DC)^36^. Samples of the diet were analyzed for dry matter (DM) by drying samples in an airforced oven at 135 °C for 2 h (method 930.15; AOAC 1990). Nitrogen was determined by the Kjeldahl method and crude protein (CP) was calculated as 6.25 × N. Ether extract (EE) was measured by the weight loss of the dry matter upon extraction with diethyl ether in a Soxhlet extraction apparatus for 8 h (method 920.85; AOAC 1990). Ca was analyzed using an atomic absorption spectrophotometer (M9W-700, Perkin-Elmer Corp., Norwalk, CT, USA) (method 968.08; AOAC 1990). The total P was analyzed by the molybdovanadate colorimetric method (method 965.17; AOAC 1990) using a spectrophotometer (UV-6100, Mapada Instruments Co., Ltd., Shanghai, China).

### Ruminal fermentation parameters

Samples of ruminal digesta were collected after slaughtered and the pH of the digesta was measured immediately using a pH metre (Model 144 PB-10, Sartorius Co., Germany). A 10-ml sample of the strained fluid was collected, acidified with 2 ml 25% (w/v) metaphosphoric acid, and stored at −20 °C for analysis of volatile fatty acids (VFA) and NH_3_-N. Volatile fatty acids in ruminal fluid were determined by gas chromatography (GC) using methyl valerate as the internal standard in an Agilent 6890 series GC equipped with a capillary column (HP-FFAP19095F-123, 30 m, 0.53 mm diameter and 1 lm thickness)^37^. Ammonia-N was assessed by the colorimetric method described by Ma *et al*.^38^. The microbial protein was measured by the trichloroacetic acid precipitation method^39^.

### DNA Extraction, amplification and Hiseq sequencing

Rumen samples were thoroughly homogenized and one millilitre of rumen fluid was centrifuged for 10 min at room temperature at 13,200 rpm. The supernatant was discarded and the remaining pellet was resuspended in 400 ml of nuclease-free water. Microbial DNA was extracted using a commercial DNA Kit (Omega Bio-tek, Norcross, GA, U.S.) according to manufacturer’s protocols. After the assessment of integrity and purity of DNA, amplification and sequencing were performed as described by Mao *et al*.^40^. The V3-V4 region of the bacteria 16S ribosomal RNA genes were amplified using primers of 338 F (5′-ACTCCTACGGGAGGCAGCAG-3′) and 806 R (5′-GGACTACHVGGGTWTCTAAT-3′) with the barcode attached. The PCR products were purified using the High Pure PCR Cleanup Micro Kit (Roche Diagnostics Ltd, Burgess Hill, West Sussex, UK) and quantified using QuantiFluor™ -ST (Promega, U.S.). Purified amplicons were pooled in equimolar and paired-end sequenced (2 × 250) on an Illumina HiSeq platform (Illumina Inc., San Diego, CA) that finished by RealBio Technology Co., Ltd.

### Data processing

The effective tags was obtained after demultiplexed, quality filtered, and analyzed using the Quantitative Insights into Microbial Ecology (QIIME, v.1.8.0) and the assembled sequences were assigned to operational taxonomic units (OTUs) at a 97% identity threshold using UPARSE^41,42^. The α diversity index, including Chao1, ACE, Shannon and Simpson were performed using QIIME^43–45^. The phylogenetic affiliation of each 16S rRNA gene sequence was analyzed by Ribosomal Database Project (RDP) Classifier (http://rdp.cme.msu.edu/) against the SILVA 16S rRNA database using confidence threshold of 70%. Principal coordinates analysis (PCoA) was used to compare treatments of samples based on unweighted Uni-Frac distance metrics^46^. Spearman correlation analysis between bacterial and ruminal fermentation parameters was performed using R corrplot.

### Statistical analysis

The growth performance, serum profiles and rumen fermentation of lambs, the alpha diversity indices, and the quantification of total bacteria were analyzed using a completely randomized design with one-way analysis of variance (ANOVA) (version 9.2, SAS Institute Inc., Cary, NC, USA). Duncan’s method for multiple comparisons was used for variables where the treatment effect was significant (*P* < 0.05), and 0.05 ≤ *P* < 0.10 was designated as a tendency. The sequencing data of this research was submitted to the Sequence Read Archive (SRA) with an accession number of SRP 162913.

## Electronic supplementary material

Figure S1
