# Supplementary material for: Influence of dietary supplementation with Bacillus licheniformis and Saccharomyces cerevisiae as alternatives to monensin on growth performance, antioxidant, immunity, ruminal fermentation and microbial diversity of fattening lambs
Source: Sci Rep. 2018 Nov 12;8:16712. doi: 10.1038/s41598-018-35081-4 (PMC6232095; doi:10.1038/s41598-018-35081-4)

Influence of dietary supplementation with *Bacillus licheniformis* and *Saccharomyces cerevisiae* as alternatives to monensin on growth performance, antioxidant, immunity, ruminal fermentation and microbial diversity of fattening lambs

Peng Jia[1], Kai Cui[1], Tao Ma[1], Fan Wan[1], Wenyi Wang[2], Dong Yang[1,2], Yunfei Wang[2], Baolin Guo[3], Lifang Zhao[3], Qiyu Diao[1*]

1. Key Laboratory of Feed Biotechnology, Ministry of Agriculture and Rural Affairs, Feed Research Institute, Chinese Academy of Agricultural Sciences, Beijing 100081, China;

2. Scientific Research Institute of Agricultural and Animal Husbandry in Bayannaoer, Inner Mongolia Bayannaoer 015000, China;

3. Beijing Xindayang Technological Co., Ltd, Beijing 100081, China

***** Corresponding author. E-mail: diaoqiyu@caas.cn.

Peng Jia and Kai Cui contributed equally to this work.

Figure S1. Principal coordinate analysis (PCoA) of microbial diversity across all treatments using a unweighted UniFrac metric.

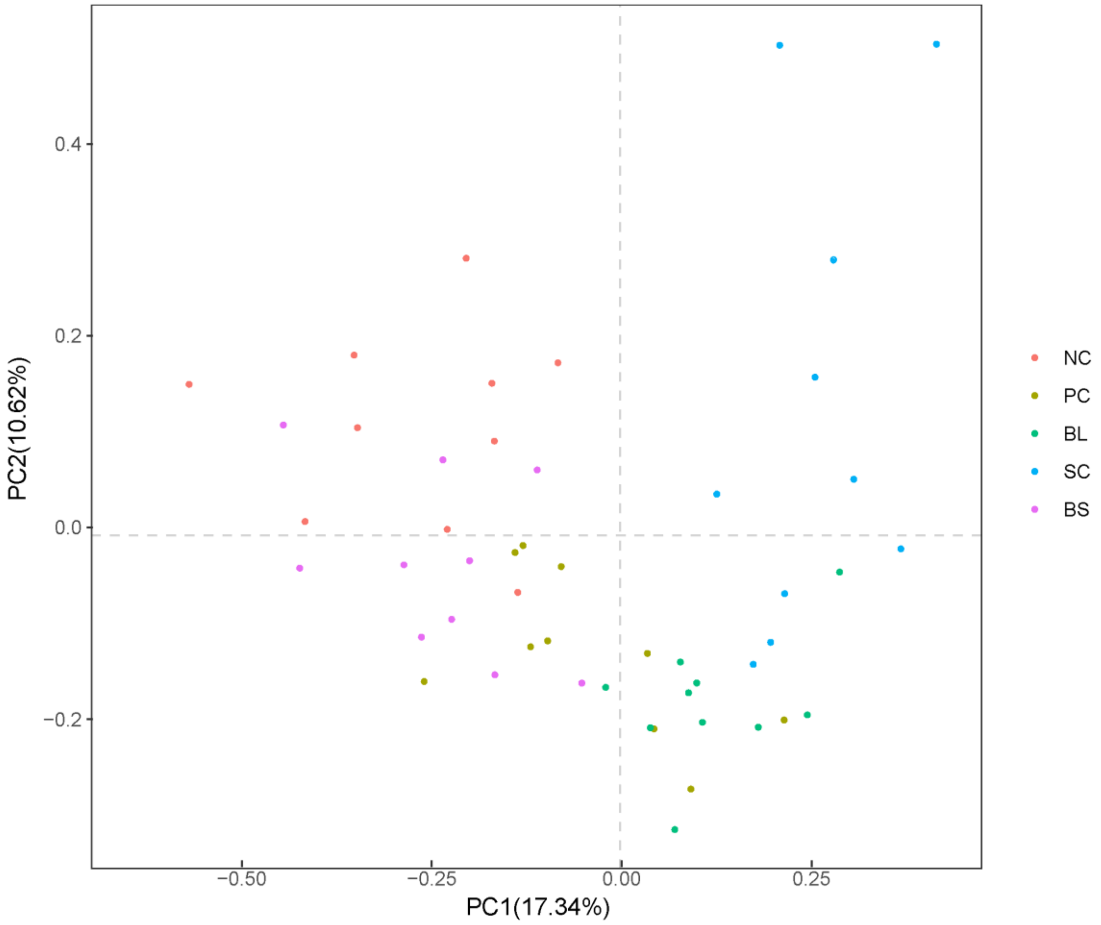

Supplement: Supplementary file 1 — Figure S1 [file 41598_2018_35081_MOESM1_ESM.pdf]
